# Protective effects of naringin against oxaliplatin-induced testicular damage in rats: Involvement of oxidative stress, inflammation, endoplasmic reticulum stress, apoptosis, and histopathology

**DOI:** 10.22038/IJBMS.2024.73824.16048

**Published:** 2024

**Authors:** Nurhan Akaras, Cihan Gür, Cuneyt Caglayan, Fatih Mehmet Kandemir

**Affiliations:** 1 Department of Histology and Embryology, Faculty of Medicine, Aksaray University, Aksaray, Turkey; 2 Department of Biochemistry, Faculty of Veterinary Medicine, Atatürk University, Erzurum, Turkey; 3 Department of Medical Biochemistry, Faculty of Medicine, Bilecik Seyh Edebali University, Bilecik, Turkey; 4 Department of Medical Biochemistry, Faculty of Medicine, Aksaray University, Aksaray, Turkey

**Keywords:** Apoptosis, Endoplasmic reticulum – stress, Inflammation, Naringin, Oxaliplatin, Testicular toxicity

## Abstract

**Objective(s):**

Oxaliplatin (OXL) is a platinum-based chemotherapeutic agent widely used in the treatment of colorectal cancer. Unfortunately, this important drug also causes unwanted side effects such as neuropathy, ototoxicity, and testicular toxicity. This study aimed to investigate the possible protective effects of naringin (NRG) against OXL-induced testicular toxicity in rats.

**Materials and Methods:**

In the present study, rats were injected with OXL (4 mg/kg, b.w./day, IP) in 5% dextrose solution 30 min after oral administration of NRG (50 and 100 mg/kg, b.w./day) on the 1st, 2nd, 5th, and 6th days. Then, the rats were sacrificed on the 7th day and the testicular tissues were removed.

**Results:**

The results showed that NRG decreased (*P*<0.001) lipid peroxidation, increased (*P*<0.001) the activities of superoxide dismutase (SOD), glutathione peroxidase (GPx), catalase (CAT), and the levels of glutathione (GSH), and also maintained the testis histological architecture and integrity. NRG decreased the levels of apoptosis-related markers such as caspase-3, Bax, and Apaf-1 and increased Bcl2 in the OXL-induced testicular toxicity (*P*<0.001). In addition, NRG reversed the changes in mRNA transcript levels of oxidative stress, inflammation, and endoplasmic reticulum stress parameters such as Nrf2, HO-1, NQO1, RAGE, NLRP3, MAPK-14, STAT3, NF-κB, IL-1β, TNF-α, PERK, IRE1, ATF6, and GRP78 in OXL-induced testicular toxicity (*P*<0.001).

**Conclusion:**

Our results demonstrated that NRG can protect against OXL-induced testicular toxicity by enhancing the anti-oxidant defense system and suppressing apoptosis, inflammation, and endoplasmic reticulum stress.

## Introduction

With a projected 20 million new occurrences worldwide, cancer is a deadly disease that affects people all over the world (1). It is a major reason for death and a significant obstacle to raising life expectancy in developed nations (2). However, metastasis and unsuccessful treatment strategies are frequently to blame for cancer deaths. Surgery, radiation, immunotherapy, hormonal therapy, and chemotherapy are the main treatment modalities. Chemotherapy’s odd advantage is that it spreads across most tissues and kills cancer cells when they metastasize (3). To target the characteristics of tumors as a treatment method, numerous chemotherapeutic drugs have previously been created, and more are being developed. Despite notable advancements in treatment methods to date, chemotherapeutic failure is caused by unfavorable effects, toxicities brought on by anticancer medications, or the emergence of multi-drug resistance. However, platinum-based antineoplastic medicines are frontline anticancer treatments utilized in various cancers, including testicular, lung, colon, and gastric cancers today (4, 5). Third-generation platinum-based anticancer drug oxaliplatin (OXL) is more effective and less hazardous than cisplatin and carboplatin. The inescapable side effects of OXL have been reported with its expanding therapeutic use. Peripheral neurotoxicity, myelosuppression, and gastrointestinal disturbances are the main side effects of OXL (6, 7).

One method to decrease the negative effects of OXL in medical practice is to protect the tissues against undesirable side effects. Therefore, it may be helpful to use natural substances with anti-oxidant properties as therapeutic agents since they could lower the severity of OXL-induced toxicities. Anti-oxidants are frequently consumed as nutrients, and extensive research has been done on how well they work to reduce the toxicity of various medications on cells, tissues, and organs (8-10). The natural flavanone naringin (NRG) has a variety of biological and pharmacological functions. Growing body of data indicates that NRG can modulate both acute and long-term inflammatory responses, suggesting its therapeutic potential. NRG is indeed useful in managing a number of inflammation-related disorders, including sepsis, acute hepatitis, fibrosis, and cancer (11-13). It possesses anti-cancer, anti-inflammatory, anti-oxidant, and anti-proliferative properties from a pharmaceutical perspective (14-16). 

Despite a sizable body of research supporting NRG’s protective effects against a number of toxic compounds, its protective effects against OXL-induced testicular toxicity have not been studied. Therefore, the goal of the current study is to determine how well NRG protects rats’ testicles from OXL-induced damage by reducing oxidative stress, inflammation, apoptosis, and endoplasmic reticulum stress.

## Materials and Methods

***Drugs and chemicals***

Oxaliplatin was purchased from DEVA Company (Istanbul, Turkey) as oxalpine (100 mg/20 ml injectable solution). All other chemicals, including NRG, were obtained from Sigma-Aldrich (St. Louis, MO, USA). Analyses of apoptotic markers (caspase-3, Bax, and Bcl-2) were performed with commercial kits purchased from YL Biont (Shanghai, China), according to the procedure specified by the manufacturer.

***Animals and ethical approval***

Thirty-five male *Sprague Dawley* rats (12–13 weeks of age; weighing 230-250 g) were obtained from the Experimental Animal Research Center of Atatürk University (Erzurum, Turkey). Animals were housed in standard conditions of temperature, humidity, and dark/light cycle. The animals were fed standard pellet feed and tap water was provided *ad libitum*. Before the treatment, animals were left for 7 days to acclimatize. All procedures involving the animals were designed in accordance with the Animal Experiments Local Ethics Committee of Atatürk University (Approval No: 2021-03/98).

***Experimental design***

Animals were divided into 5 groups, each group consisted of 7 rats.

I) Control group: 5% dextrose solution was administered intraperitoneally to the animals in this group on the 1st, 2nd, 5th, and 6th days.

II) NRG group: Animals were administered orally by gavage NRG (100 mg/kg b.w.) on the 1st, 2nd, 5th, and 6th days (17).

III) OXL group: OXL was dissolved in 5% dextrose solution as 4 mg/kg b.w. and given intraperitoneally on the 1st, 2nd, 5th, and 6th days (total cumulative dose is 16 mg/kg b.w.) (18).

IV) OXL + NRG-50 group: NRG was dissolved in distilled water as 50 mg/kg b.w. and given orally by gavage on the 1st, 2nd, 5th, and 6th days. 

30 min after NRG administration, OXL was dissolved in 5% dextrose solution as 4 mg/kg b.w. and administered intraperitoneally (total cumulative dose is 16 mg/kg/b.w.).

V) OXL + NRG-100 group: NRG was dissolved in distilled water as 100 mg/kg b.w. and given orally by gavage on the 1st, 2nd, 5th, and 6th days. 30 min after NRG administration, OXL was dissolved in 5% dextrose solution as 4 mg/kg b.w. and administered intraperitoneally (total cumulative dose is 16 mg/kg/b.w.).

On the seventh day, the animals were anesthetized by inhalation of sevoflurane, and the animals were sacrificed. One of the testicular tissues taken from the animals was first washed with physiological saline and dried with a paper towel. Then the tissue was taken into a plastic petri dish and stored at -80 °C. After the tissues were stored in the refrigerator for approximately 2-3 months, they were ground in liquid nitrogen for biochemical analysis and homogenized in the appropriate buffer on ice with a homogenizer (Tissuelyser II, Qiagen). In our previous study, the details of the homogenization process were explained (19). The other testicular tissue was fixed with %10 formalin for histopathological analysis.

***Oxidative stress markers in testis tissue***

The superoxide dismutase (SOD) activity was determined by the method described by Sun *et al.* (20). Catalase (CAT) activity was assessed by the method of Aebi (21). Glutathione peroxidase (GPx) activity was determined by the method described by Lawrence and Burk (22). The assay of glutathione (GSH) level was done according to the method of Sedlak and Lindsay (23). The Lipid peroxidation was evaluated by the measurement of malondialdehyde (MDA) according to the method of Placer *et al*. (24). Protein content in the testis tissue was measured according to Lowry *et al*. (25).

***Detection of caspase-3, Bax, and Bcl-2 levels in testicular tissue by ELISA method***

In order to determine the cysteine-aspartic acid protease (Caspase-3), BCL2 associated x (Bax), and B-cell lymphoma 2 (Bcl-2) levels, the testis tissue was homogenized in phosphate buffer (pH 7.4, 0.1 M) via a tissue lyser instrument (TissueLyser II, Qiagen). It was then centrifuged at 4 °C (3000 rpm for 30 min). Supernatants from testis homogenates were used for Caspase-3, Bax, and Bcl-2 analysis. Briefly, reagents, samples, and standards are first prepared. The prepared samples are then incubated with antibodies (biotin and HRP) at 37 °C for 60 min. The washing process is performed 5 times with washing solution, chromogen A and B solutions are added and incubated at 37 °C for 10 min. Afterwards, a stop solution is added and the measurement is made. The plates were read at 450 nm with an ELISA microplate reader (Bio-Tek, Winooski, VT, USA).

***RT-PCR analysis***

The mRNA transcript levels of Nuclear factor erythroid 2-related factor 2 (Nrf-2), Heme Oxygenase 1 (HO-1), NAD(P)H Quinone Dehydrogenase 1** (**NQO1), Receptor for Advanced Glycation Endproducts** (**RAGE), NLR Family Pyrin Domain Containing 3 (NLRP3) Apoptotic protease-activating factor 1 (Apaf-1), Mitogen-Activated Protein Kinase 14 (MAPK14), Signal transducer and activator of transcription-3 (STAT-3), Nuclear Factor kappa B (NF-κB), Tumor necrosis factor-alpha (TNF-α), Interleukin-1 beta (IL-1β), Activating transcription factor 6 (ATF-6), Protein Kinase RNA-Like ER Kinase (PERK), Inositol-requiring enzyme 1 (IRE1), and Glucose-regulated protein 78 (GRP-78) genes were analyzed. For this, testicular tissues were first powdered with liquid nitrogen, and then total RNA was isolated with QIAzol Lysis Reagent (79306; Qiagen) according to the manufacturer’s instructions. After the treatments, the concentrations of RNAs were measured in the NanoDrop (BioTek Epoch) device. Then, RNAs from total RNAs (RNA concentrations of all groups were equalized to the concentration specified in the kit) were converted into cDNAs with a iScript cDNA Synthesis Kit (Bio-Rad). The obtained cDNAs were used to determine the mRNA transcript levels of the genes whose sequences are given in Table 1. For this, iTaq Universal SYBR Green Supermix (BIORAD) was used and analyses were performed on Rotor-Gene Q (Qiagen). β-Actin was used as the housekeeping gene and fold changes were calculated by the 2^-∆∆CT ^method (26).

***Histopathological analysis***

Testicular tissues were fixed in a 10% formalin solution for 48 hr. In order to follow the fixed tissues, they were first washed in tap water for 6–8 hr and dehydrated by passing through increasing-grade alcohol. After passing through xylene to make the tissues transparent, they were treated with paraffin, and then paraffin blocks were prepared. Sections of 5 μm were taken from paraffin blocks with the help of a microtome. Sections were stained with Hematoxylin–Eosin (H&E) in accordance with the procedures for histological evaluation. Stained tissues were examined under a light microscope (Olympus Cx43; Japan) and then photographed. The Johnsen scoring system was used to evaluate the germinal epithelial cell organization of the seminiferous tubules and the density of Sertoli cells in the testis (27). Twenty seminiferous tubules were examined in each section and the average was taken for each section. Evaluations were performed in a blinded manner using scores between 1 and 10 according to the score. The scoring criteria are as follows:

1. Tubular sclerosis (no cells in the seminiferous tubule lumen), 2. No germinal cells, only Sertoli cells, 3. only spermatogonia, 4. No spermatozoa and spermatids, less than 5 spermatocytes, 5. No spermatozoa, no spermatids, spermatocytes present, 6. No spermatozoa, no late spermatids, less than 10 early spermatids, 7. No spermatozoa, lots of spermatids, 8. Organized germinal epithelium (multilayered), but <10 spermatozoa in lumen, 9. Although germinal epithelium is seen, an insignificant amount of debris may obstruct the lumen, spermatozoa, and rash epithelium in the occluded lumen., 10. Organized germinal epithelium (stratified germinal epithelium around the central lumen), numerous spermatozoa.

***Statistical analysis***

All biochemical **and histological** data were analyzed using one-way ANOVA with Tukey’s post hoc tests for multiple comparisons. GraphPad Prism 5.0 software was used for the data analysis. The results are presented as the mean ± standard deviation (SD). The values of *P*<0.05 were statistically significant.

## Results

***Protective effects of NRG on OXL-induced oxidative stress in testis tissue***

The effect of NRG on the anti-oxidant enzyme activities in testis homogenates of rats treated with OXL is shown in Table 2. Administration of OXL caused a significant decrease (*P*<0.001) in the activities of SOD, CAT, and GPx in the testis compared to the control group. However, administration of NRG (50 and 100 mg/kg) significantly reduced oxidative damage by increasing the above-mentioned enzyme activities compared to the OXL-treated group (*P*<0.001). When NRG high-dose treatment was compared to low-dose NRG treatment, MDA levels were determined to be lower and SOD, CAT, and GPx activities were higher (*P*<0.001). 

The content of GSH in rats injected with OXL decreased (*P*<0.001) significantly, whereas the level of MDA increased significantly compared to those in the control group (*P*<0.001). In contrast, NRG significantly increased the content of GSH and decreased the level of MDA in groups 4 and 5 (OXL+NRG-50 and OXL+NRG-100) as compared to the OXL-treated group (*P*<0.001) Table 2. It was observed that the GSH level of the OLX+NRG-100 group was higher than the OLX+NRG-50 group (*P*<0.001). Additionally, there is no significant difference between the control and NRG groups in terms of MDA, SOD, CAT, GPx, and GSH markers.

**Figure 1:**
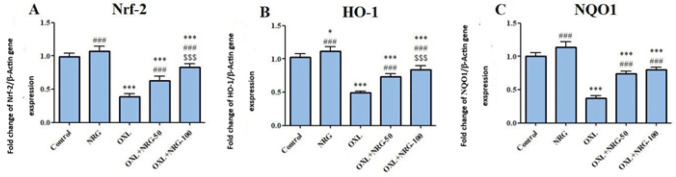
Protective effects of naringin (NRG) and oxaliplatin (OXL) treatments on nuclear factor erythroid 2-related factor 2 (Nrf-2), Heme oxygenase 1 (HO-1) and NAD(P)H Quinone Dehydrogenase 1 (NQO1) mRNA expression levels in testis tissue

To investigate the protective effect of NRG on OXL-induced testicular oxidative stress, we investigated the mRNA transcript levels of Nrf2, HO-1, and NQO1. Our qRT-PCR results showed that OXL significantly (*P*<0.001) decreased the mRNA transcript levels of Nrf2 (*P*<0.001), HO-1 (*P*<0.001), and NQO1 (*P*<0.001). However, administration of NRG resulted in a significant increase in the levels of these parameters (*P*<0.001). Additionally, when the OXL+NRG 100 group was compared to the OXL+NRG 50 group, Nrf2, and HO-1 levels were higher NQO1 (*P*<0.001), but no difference was observed in terms of NQO1 (Figure 1A-C).

***Protective effects of NRG on OXL-induced inflammation in testis tissue***

**Figure 2:**
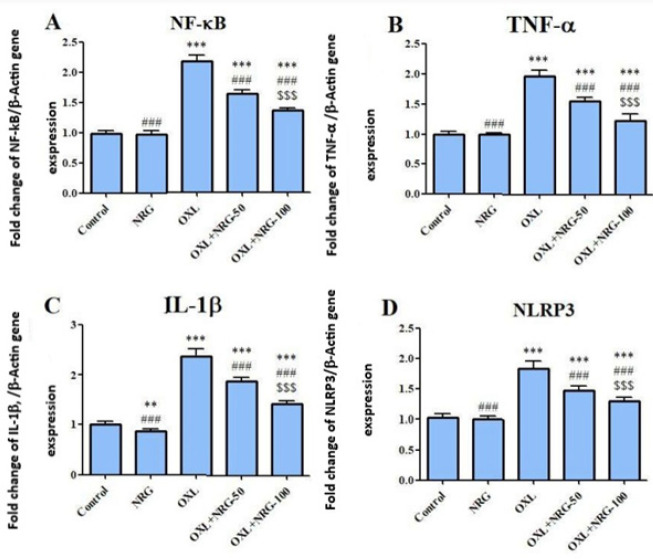
Protective effects of naringin (NRG) and oxaliplatin (OXL) treatments on nuclear factor kappa B (NF-κB), tumor necrosis factor-α (TNF-α), interleukin-1 beta (IL-1β) and NLR Family Pyrin Domain Containing 3 (NLRP3) mRNA expression levels in testis tissue

To ascertain inflammation in testis tissue, mRNA transcript levels of NF-κB, TNF-α, IL-1β, and NLRP3 genes were evaluated. Our data demonstrated that the mRNA expression levels of NF-κB, TNF-α, IL-1β, and NLRP3 were markedly increased in the testis of the OXL-induced rats **(***P*<0.001). Treatment with NRG (50 and 100 mg/kg) decreased the levels of these inflammatory biomarkers (*P*<0.001). In addition, the levels of NF-κB, TNF-α, IL-1β, and NLRP3 were lower in the OXL+NRG 100 group compared to the OXL+NRG 50 group (*P*<0.001). (Figure 2A-D). 

***Protective effects of NRG on OXL-induced apoptosis in testis tissue***

**Figure 3:**
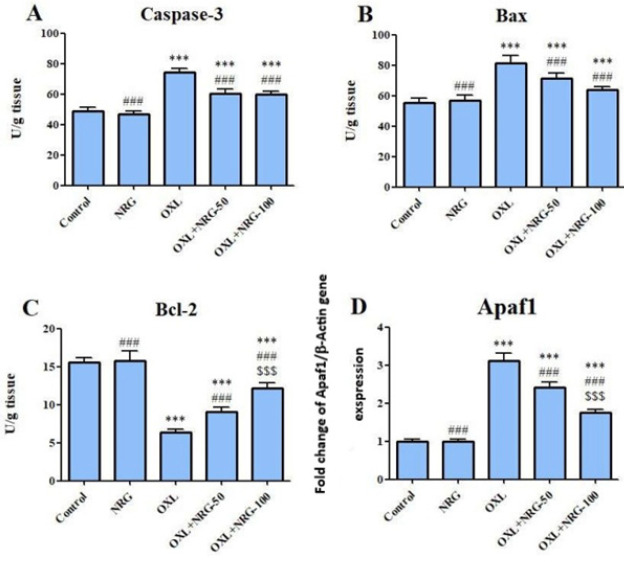
Protective effects of naringin (NRG) and oxaliplatin (OXL) treatments on cysteine–aspartic acid protease (caspase-3), Bcl-2 associated X protein (Bax) and B-cell lymphoma-2 (Bcl-2) levels, and Apoptotic peptidase activating factor 1 (Apaf-1) mRNA expression levels in testis tissue

The effects of NRG and/or OXL on caspase-3, Bax, and Bcl2 levels and mRNA levels of Apaf-1 in testicular tissue are presented in Figure 3A-D. OXL caused a significant (*P*<0.001) increase in the levels of caspase-3, Bax, and Apaf-1 and a (*P*<0.001) decrease in the level of Bcl-2 compared to the control group. However, NRG administration significantly reduced apoptosis by reversing the levels of these parameters compared to the OXL-treated group (*P*<0.001). In addition, the levels of Bcl2 and Apaf 1 were lower in the OXL+NRG 100 group compared to the OXL+NRG 50 group (*P*<0.001). When comparing NRG and the control group, there is no difference in terms of apoptosis parameters.

***Effect of NRG on the STAT3 inflammation pathway in OXL-induced testis tissue***

**Figure 4:**
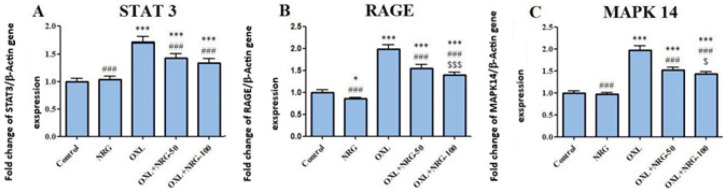
Protective effects of naringin (NRG) and oxaliplatin (OXL) treatments on Signal transducer and activator of transcription 3 (STAT3), Receptor for Advanced Glycation Endproducts (RAGE) and mitogen-activated protein kinase 14 (MAPK14) mRNA expression levels in testis tissue

When compared to the control group, OXL injection significantly (*P*<0.001) increased mRNA levels of STAT3, RAGE and MAPK14 while their expression was down-regulated in the OXL+NRG 50 and OXL+NRG 100 groups (*P*<0.001) (Figure 4A-C). High-dose NRG treatment (OXL+NRG 100) was found to be significantly lower in terms of RAGE (*P*<0.001 and MAPK14 (*P*<0.05) levels than low-dose NRG treatment (OXL+NRG 50).

***Protective effects of NRG on OXL-induced endoplasmic reticulum stress in testis tissue***

**Figure 5:**
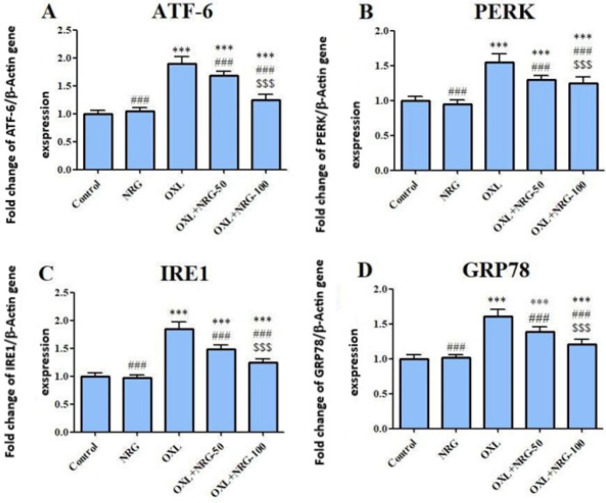
Protective effects of naringin (NRG) and oxaliplatin (OXL) treatments on activating transcription factor-6 (ATF-6), protein kinase (PKR)-like ER kinase (PERK), Inositol-requiring enzyme-1 (IRE1) and Glucose-regulated protein 78 (GRP78) mRNA expression levels in testis tissue

The expression profiles of ATF-6, PERK, IRE1, and GRP78 genes in the testis were determined with qRT-PCR in the control and treatment groups. When compared to the control group, OXL injection significantly (*P*<0.001) elevated mRNA levels of ATF-6, PERK, IRE1, and GRP78 while their expression was down-regulated in the OXL+NRG 50 and OXL+NRG 100 groups (*P*<0.001). It was observed that OLX+NRG 100 treatment was more significant in reducing ATF-6, PERK, IRE1, and GRP78 levels compared to OLX+NRG 50 treatment (*P*<0.001). (Figure 5A-D).

***Histopathological results***

**Figure 6:**
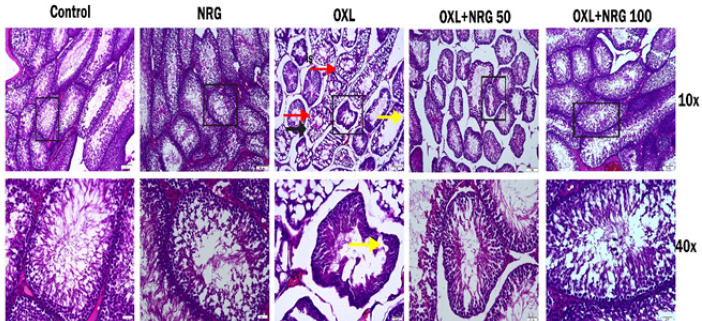
Photomicrographs of testis tissue histological changes

**Figure 7:**
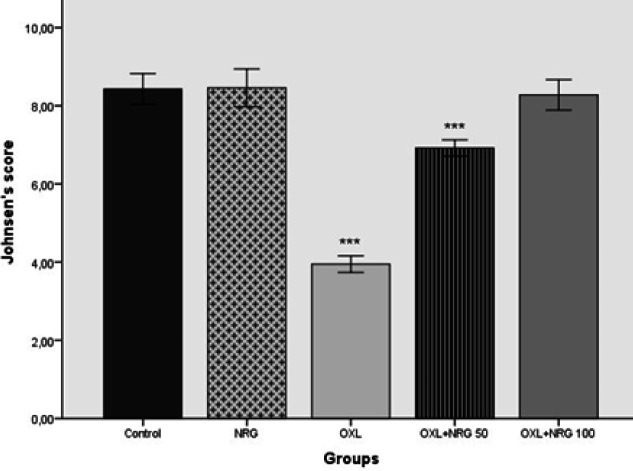
Effects of naringin (NRG) and oxaliplatin (OXL) treatments on Johnsen Testicular Biopsy Score

Histological changes in testicular tissues stained with Hematoxylin&Eosin (H&E) are shown in Figure 6. When the sections in both control and NRG groups were examined, the seminiferous tubules, germinal epithelium, and interstitial area had normal morphological appearance. The germ cell architecture looked neatly ordered. Also, the lumen appeared to be filled with tailed spermatids. However, in the OXL group, shedding of the germinal epithelium, disruption of the basement membrane, vacuolization in the interstitial area, and germ cell arrest in the spermatogenesis cell division series were remarkable. Most of the seminiferous tubules were atrophic. Degenerative changes were less evident in the OXL+NRG 50 and OXL+NRG 100 groups. It was observed that the tubule lumen in the OLX+NRG 100 group was filled with tailed spermatid cells. Johnsen score results of the groups are shown in Figure 7. When the Johnsen scores of the groups were compared, there was no statistical significance between the control, NRG, and OXL+NRG 100 groups. When the Johnsen score results of the OXL group were compared with the other groups, there was a statistically significant difference and lower than the other groups (*P*<0.001). It was also seen that the score result of the OXL+NRG 100 group was higher than that of the OXL+NRG 50 group (*P*<0.001).

**Table 1:** The primer sequences of the related genes used in the RT-PCR (R: Reverse; F: Forward)

**Gene**	**Sequences (5’-3’)**	**Length (bp)**	**Accession No**
**Nrf2**	F: TTTGTAGATGACCATGAGTCGC R: TCCTGCCAAACTTGCTCCAT	161	NM_031789.2
**HO-1**	F: ATGTCCCAGGATTTGTCCGA R: ATGGTACAAGGAGGCCATCA	144	NM_012580.2
**NQO1**	F: CTGGCCAATTCAGAGTGGCA R: GATCTGGTTGTCGGCTGGAA	304	NM_017000.3
**RAGE**	F: CTGAGGTAGGGCATGAGGATG R: TTCATCACCGGTTTCTGTGACC	113	NM_053336.2
**NLRP3**	F: TCCTGCAGAGCCTACAGTTG R: GGCTTGCAGCACTGAAGAAC	185	NM_001191642.1
**Apaf-1**	F: ACCTGAGGTGTCAGGACC R: CCGTCGAGCATGAGCCAA	192	NM_023979.2
**MAPK14**	F: GTGGCAGTGAAGAAGCTGTC R: GTCACCAGGTACACATCGTT	170	NM_031020.2
**STAT3**	F: TACCTGGAGCAGCTTCATCA R: GATCTCGCCCAAGAGGTTAT	153	NM_012747.2
**NF-** **B**	F: AGTCCCGCCCCTTCTAAAAC R: CAATGGCCTCTGTGTAGCCC	106	NM_001276711.1
**TNF-**	F: CTCGAGTGACAAGCCCGTAG R: ATCTGCTGGTACCACCAGTT	139	NM_012675.3
**IL-1**	F: ATGGCAACTGTCCCTGAACT R: AGTGACACTGCCTTCCTGAA	197	NM_031512.2
**ATF-6**	F: TCAACTCAGCACGTTCCTGA R: GACCAGTGACAGGCTTCTCT	130	NM_001107196.1
**PERK**	F: GATGCCGAGAATCATGGGAA R: AGATTCGAGAAGGGACTCCA	198	NM_031599.2
**IRE1**	F: GCAGTTCCAGTACATTGCCATTG R: CAGGTCTCTGTGAACAATGTTGA	163	NM_001191926.1
**GRP78**	F: CATGCAGTTGTGACTGTACCAG R: CTCTTATCCAGGCCATATGCAA	143	NM_013083.2
**-Actin**	F: CAGCCTTCCTTCTTGGGTATG R: AGCTCAGTAACAGTCCGCCT	360	NM_031144.3

**Table 2.** The effect of naringin (NRG) treatment on oxaliplatin (OXL)-induced testis tissue on malondialdehyde (MDA), glutathione (GSH) levels, catalase (CAT), superoxide dismutase (SOD) and glutathione peroxidase (GPx) activities in rats. Control, naringin (NRG), oxaliplatin (OXL), OXL+NRG-50, OXL+NRG-100. All data were expressed as mean ± standard deviation (SD)

**Parameters**	** Control**	** NRG**	** OXL**	**OXL+NRG-50**	**OXL+NRG-100**
**MDA(nmol/g tissue)**	28.90±1.92	28.49±2.06^###^	57.63±2.17^***^	46.62±1.94^***/###^	35.65±1.87^***/###/$$$^
**GSH (nmol/g tissue)**	5.35±0.09	5.30±0.10^###^	3.50±0.14^***^	4.17±0.13^***/###^	4.47±0.14^***/###/$$$^
**CAT (katal/g protein)**	9.03±0.70	9.15±0.65^###^	3.95±0.52^***^	5.79±0.49^***/###^	6.77±0.63^***/###/$$$^
**SOD (U/g tissue)**	15.25±0.63	15.78±0.69^###^	5.94±0.49^***^	8.63±0.68^***/###^	11.01±0.66^***/###/$$$^
**GPx (U/g tissue)**	18.80±1.11	17.73±0.84^###^	7.96±0.67^***^	10.99±0.79^***/###^	12.41±0.64^***/###/$$$^

Statistical significance (Control vs others: ***P<0.001, OXL vs others: ###*P*<0.001, OXL+NRG-50 vs OXL+NRG-100: $$$*P*<0.001) was analyzed using One Way ANOVA

Control, naringin (NRG), oxaliplatin (OXL), OXL+NRG-50, OXL+NRG-100

(A) Fold change of Nrf-2 gene expression. (B) Fold change of HO-1 gene expression. (C) Fold change of NQO1gene expression. (Control vs others: *P<0.05, ***P<0.001, OXL vs others: ###*P*<0.001, OXL+NRG-50 vs OXL+NRG-100: $$$*P*<0.001) was analyzed using One-way ANOVA).

Control, naringin (NRG), oxaliplatin (OXL), OXL+NRG-50, OXL+NRG-100

(A) Fold change of Nf-kB gene expression. (B) Fold change of TNF-α gene expression. (C) Fold change of IL-1β gene expression. (D) Fold change of NLRP3 gene expression. (Control vs others: **P<0.01, ***P<0.001, OXL vs others: ###*P*<0.001, OXL+NRG-50 vs OXL+NRG-100: $$$*P*<0.001) was analyzed using One-way ANOVA).

Control, naringin (NRG), oxaliplatin (OXL), OXL+NRG-50, OXL+NRG-100

(A) Represent the levels of caspase-3. (B) Represent the levels of Bax. (C) Represent the levels of Bcl-2. (D) Fold change of Apaf-1 gene expression. (Control vs others: ***P<0.001, OXL vs others: ###*P*<0.001, OXL+NRG-50 vs OXL+NRG-100: $$$*P*<0.001) was analyzed using One-way ANOVA).

Control, naringin (NRG), oxaliplatin (OXL), OXL+NRG-50, OXL+NRG-100

(A) Fold change of STAT3 gene expression. (B) Fold change of RAGE gene expression. (C) Fold change of MAPK 14 gene expression. (Control vs others: *P<0.05, ***P<0.001, OXL vs others: ###*P*<0.001, OXL+NRG-50 vs OXL+NRG-100: $*P*<0.05, $$$*P*<0.001) was analyzed using One-way ANOVA).

Control, naringin (NRG), oxaliplatin (OXL), OXL+NRG-50, OXL+NRG-100

(A) Fold change of ATF-6 gene expression. (B) Fold change of PERK gene expression. (C) Fold change of IRE1 gene expression. (D) Fold change of GRP78 gene expression. (Control vs others: ***P<0.001, OXL vs others: ###*P*<0.001, OXL+NRG-50 vs OXL+NRG-100: $$$*P*<0.001) was analyzed using One-way ANOVA).

(H&E staining) Control group; naringin (NRG) group; oxaliplatin (OXL) group; is: intersisyel space, red arrow: vacuole, black arrow: germinal epithelial damage, yellow arrow: germ cell loss; OXL+NRG-50 group; OXL+NRG-100 group

Control, naringin (NRG), oxaliplatin (OXL), OXL+NRG-50, OXL+NRG-100. *** *P * < 0.001, compared with the control group. Data were tested using ANOVA followed by Tukey test. Values are mean ± SD

## Discussion

A growing body of evidence has demonstrated that the toxicity of platinum-based antineoplastic drugs on delicate body organs is related to free radical formation, DNA damage, oxidative injury, apoptosis, and endoplasmic reticulum stress (1). One of the side effects of the OXL is testicular damage (28). The current study projected ameliorative and preventative effects of NRG on testis toxicity induced by a platinum-based antineoplastic drug, OXL.

We examined oxidative stress indicators to shed some light on the molecular mechanisms underpinning the protective effects of NRG on OXL-induced testicular toxicity. A significant factor in the pathophysiology of OXL is oxidative stress (29). Increasing concentrations of O^−2^, H_2_O_2_, and OH^-^ radicals are anticipated to act as mediators of oxidative damage brought on by OXL. It is mostly characterized by high levels of free radicals and a compromised anti-oxidant defense system, resulting in a redox imbalance (30). In the current study, the rats treated with OXL had significantly higher levels of MDA, a byproduct of lipid peroxidation, than animals in the untreated control group. The present work assessed the levels of host anti-oxidant system-related enzymes and molecules, such as SOD, GPx, and total GSH, in addition to oxidative stress indicators like MDA. Due to its anti-oxidant capacity, SOD is a crucial enzyme in the host’s defense mechanism against oxidative stress (31). Several studies have demonstrated that severe oxidative stress weakens anti-oxidant defenses by damaging enzymes like SOD and GPx (31, 32). Additionally, GSH and GPx are crucial components of natural cell defense mechanisms against oxidative stress. H_2_O_2_ is converted into water by the anti-oxidant enzyme GPx, and GSH is a crucial cofactor for GPx to work properly in this reaction (33). Low levels of GPx and GSH have been linked to diseases like cancer, chronic illness, and aging that are caused by free radicals (34). Recently, it has been reported that GSH is a potential medication for preventing OXL-induced toxicity without materially impairing OXL’s therapeutic effectiveness. It was believed that the avoidance of the accumulation of platinum metabolites was the mechanism underlying glutathione’s protective activity (35,36). Therefore, NRG’s ameliorative benefits may be due to its ability to stop the decreases in SOD, GPx, and GSH levels brought on by OXL. In a previous study, NRG was shown to significantly reduce MDA levels and increase SOD, CAT, and GPx activities in methotrexate-induced testicular tissue of rats (37).

Further research revealed that Nrf2 activation, which triggers phase II detoxifying and anti-oxidant enzymes, is related to the anti-oxidant effects of NRG in OXL-induced testes injury. One of the key regulators of the cytoprotective and detoxifying genes that reduce oxidative damage is Nrf2 (38). Nrf2 interacts with Keap1 under normal physiological circumstances (39). After being exposed to oxidative injury, Nrf2 separates from Keap1 and then increases the transcription of numerous anti-oxidant response element-controlled genes. This causes the expression of several gene encoding anti-oxidant and phase II drug-detoxifying enzymes, such as HO-1 and NQO1, to be induced. The Nrf2 signaling pathway is activated to diminish oxidative damage and the production of pro-inflammatory cytokines (40). DNA transcription, cytokine production, and cell survival are all controlled by NF-κB. NF-κB is present in almost all animal cell types and is involved in how cells respond to stress, cytokines, free radicals, heavy metals, and UV radiation. NF-κB activation causes production and recruitment of pro-inflammatory cytokines (TNF-α, IL-1β, and IL-6) (41). According to the findings of the study, a decrease in testicular Nrf2, HO-1, and NQO1 gene expression was observed in OXL-treated rats.

Two commonly known Nrf2 downstream target genes, HO-1 and NQO1, as well as the testicular Nrf2 gene, were all considerably up-regulated by NRG therapy. These results suggest that NRG decreases OXL-induced testicular toxicity and oxidative damage *in vivo* by activating the Nrf2 pathway. According to reports, NRG inhibits the NF-κB signaling pathway to effectively reduce inflammation. NRG can reduce the expression of several inflammatory markers, including toll-like receptor 4 (TLR4), TNF-α, IL-1β, IL-6, iNOS, and COX-2, by weakening the NF-κB pathway and activating the AMP-activated protein kinase (AMPK), which is linked to the inhibition of a number of pro-inflammatory signaling pathways (17, 42, 43). The NLRP3 inflammasome is a crucial part of the innate immune system that promotes caspase-1 activation and the release of pro-inflammatory cytokines IL-1 and IL-18 in reaction to microbial infection and cellular injury. The abnormal activation of the NLRP3 inflammasome has been connected to several inflammatory diseases, such as Alzheimer’s disease, diabetes, and atherosclerosis. Ionic flux, mitochondrial malfunction, and the creation of reactive oxygen species all serve in the activation of the NLRP3 inflammasome (44). According to our findings, NRG co-treatment prevented testicular damage brought on by OXL by reducing the levels of the pro-inflammatory cytokines and NLRP3 inflammasome. Therefore, the protective effect of NRG is also connected with the reduction of testicular inflammation in addition to the prevention of oxidative stress.

This study also looked at the biomolecular mechanism by which NRG treatment decreased the levels of OXL-induced apoptosis in the testes. Mitochondria is central to stress-induced apoptosis. Cytochrome c is released by the mitochondria and is an important factor that promotes apoptosis. Additionally, it is a crucial element in the beginning of the signaling cascade that leads to apoptosis (45). When cytochrome c is released, it forms a complex with Apaf1 and procaspase-9, which activates caspase-9 and then other effector molecules like caspase-3, ultimately leading to programmed cell death (46). The mitochondrial membrane contains Bcl-2, which is essential for preserving the integrity of the membrane. When exposed to damaging stimuli, Bax migrates from its normal location in the cytoplasm to the mitochondrial membrane, where it enhances the permeability of the mitochondrial outer membrane. As a result, Bcl-2 and Bax, respectively, promote and inhibit apoptosis (47). In order to induce apoptosis, it is crucial to maintain the Bcl-2/Bax ratio. According to the findings of our study, OXL treatment increased the amount of Bax and cleaved-caspase-3 and reduced the amount of Bcl-2 in comparison to the control as evidenced by ELISA data. OXL treatment also up-regulated the expression of Apaf-1. Whether cells enter the apoptotic state is determined by the Bcl-2/Bax ratio. Apoptosis is increased and Bcl-2 is suppressed if Bax is dominant. If not, Bax is suppressed and cells survive. The findings of this investigation revealed that OXL lowered the Bcl-2/Bax ratio to varied degrees in comparison to the control group and that the activation of caspase-mediated signal transduction may be a factor in how they cause apoptosis.

Cellular signal transcription factor STAT participates in the control of a broad array of cellular processes containing cell differentiation, proliferation, angiogenesis, immune responses, and inflammation in healthy cells. As part of the STAT family, STAT3 has been discovered to be implicated in a variety of disorders, such as cancer, where STAT3 is highly expressed and persistently activated, encouraging the growth and proliferation of tumors (48). RAGE, an immunoglobulin superfamily member with a recent evolutionary history, is encoded in the Class III area of the major histocompatibility complex. Only the lung exhibits high levels of RAGE expression that are readily detectable, but RAGE expression quickly rises at areas of inflammation, mostly on inflammatory and epithelial cells. RAGE activation is followed by the activation of numerous intracellular signaling agents such as the transcription factor NF-κB and MAP kinases (49). In a relatively recent research, histo-pathological damage was observed in the testes of mice fed an AGE-rich diet. There was a related increase in the level of RAGE, accompanied by raised MDA levels (50). In a different study, RAGE was shown to be elevated in the testes of diabetic rats (51). All eukaryotic cells have MAPKs, which transmit information to the nucleus. In many cells, MAPK14, sometimes referred to as p38, is a crucial regulator of the inflammatory response (52). In recent studies, it has been reported that MAPK 14 levels increase organ damage (53-55). Our results also show that the levels of STAT3, RAGE, and MAPK14 increased in testicular injury, while NRG administration protected the cells against this toxicity. Together, these results provided more evidence for NRG’s ability to reduce the inflammatory effects caused by OXL through down-regulating STAT3, RAGE, and MAPK14.

Accumulated unfolded/misfolded proteins cause endoplasmic reticulum stress in response to oxidative stress (56). Endoplasmic reticulum stress normally serves to protect cells by inhibiting protein synthesis and causing the creation of molecular chaperones, whereas excessive endoplasmic reticulum stress results in CHOP-associated cell apoptosis (57, 58). As demonstrated above, OXL enhanced the mRNA transcript levels of endoplasmic reticulum stress indicators ATF6, PERK, IRE1, and GRP78. It should be emphasized that the reactive oxygen species scavenging effect of NRG reversed the activation of the endoplasmic reticulum stress cascade brought on by OXL.

Histopathological examination of the testis is very important to determine male reproductive injury by OXL-induced. When testis tissues were examined histopathologically, shedding of the germinal epithelium, disruption of the basement membrane, vacuolization in the interstitial area and germ cells, and arrest in the spermatogenesis cell division series were remarkable OXL-induced testis damages. The histopathological finding of our study was that NRG treatment contributed to the recovery of testis architecture induced by OXL.

## Conclusion

Our findings demonstrated that OXL may cause testis damage through various mechanisms, such as oxidative stress, inflammation, apoptosis, and histopathological defects. NRG significantly ameliorated oxidative stress, inflammation, apoptosis, and histological changes, induced by OXL.

## Authors’ Contributions

C G, N A, and FMK designed the research. N A, C C, and C G conducted experiments. N A and FM K analyzed data. CC, NA, and FMK wrote the manuscript. All authors have read and approved the final version for publication.

## Funding

No funding was received.

## Conflicts of Interest

The authors declare that they have no conflicts of interest.
